# Specific properties of shRNA-mediated CCR5 downregulation that enhance the inhibition of HIV-1 infection in combination with shRNA targeting HIV-1 *rev*

**DOI:** 10.1007/s11033-022-07899-9

**Published:** 2022-09-12

**Authors:** Maria E. Cardona, Jorma Hinkula, Kristin Gustafsson, Birger Christensson, Britta Wahren, Abdalla J. Mohamed, C. I. Edvard Smith, H. Jose Arteaga

**Affiliations:** 1 Department of Laboratory Medicine, Clinical Research Centre, Karolinska Institutet, Karolinska, Sweden; 2 Department of Biomedical and Clinical Sciences, Linköping University, Linköping, Sweden; 3 Department of Laboratory Medicine, Division of Pathology, Karolinska University Hospital, Huddinge, Sweden; 4 Department of Microbiology, Tumor and Cell Biology, Karolinska Institutet, Karolinska, Sweden; 5 Department of Basic Science, Faculty of Health, Universidad Industrial de Santander, Santander, Colombia

**Keywords:** RNA interference, HIV-1, *Rev* gene, CCR5 receptor

## Abstract

Treatment with RNAi against HIV-1 transcripts efficiently inhibits viral replication but induces selection of escape mutants; therefore, the CCR5 coreceptor was suggested as an additional target. Blocking viral and host transcripts improved the antiviral effect. We have used short hairpin RNA (shRNA) targeting the human CCR5 (shCCR5) or the HIV-1 rev (shRev) transcripts to demonstrate distinctive properties of anti-*CCR5* shRNA: shCCR5 induced more sustained protection than shRev; partial reduction in CCR5 expression substantially decreased HIV-1 infection, and shCCR5 performed better than shRev in the mixed shRNA-treated and untreated cultures. These observations indicate that CCR5 inhibitors should be conveniently included in HIV-1 gene silencing treatment schedules when only a certain cell fraction is protected to further reduce endogenous virus in a properly ART-treated HIV-1 infected individual.

## Introduction

RNA interference (RNAi) mediates sequence-specific degradation of RNA transcripts. Improved RNAi technologies and new gene editing tools, such as CRISPR-Cas9, hold great potential for the treatment of HIV-1 infection [1, 2]. In fact, it has been demonstrated that RNAi efficiently inhibits viral replication by targeting HIV-1 RNA sequences [1] or viral infection by impairing CCR5 expression [3]. *Rev* is an interesting viral target because the Rev gene/protein interactions are among the most functionally conserved features of the HIV-1 genome, since indispensable RNA-RNA and RNA-protein interactions occur during progenomic transport and virion production [4]. Even though other HIV-1 genome targets have proven to work similarly well, RNAi readily selects escape mutants. Viral escapes can be limited to a great extent, though not completely, by targeting multiple highly conserved regions of the viral genome [5]. To further reduce viral RNAi-escape mutants, the CCR5 coreceptor was suggested as an additional target [3], since its homozygous 32-bp deletion mutant (CCR5Δ32) protects from CCR5-dependent HIV-1 variants [6]. A truly cured HIV-infected patient received a bone marrow transplant from a CCR5Δ32 homozygous donor followed by discontinuation of antiretroviral therapy [7]; additionally, in another patient, similarly treated, HIV-1 remission has been maintained over two years [8]. However, the virus is still able to switch its affinity to other coreceptors such as CXCR4 [9, 10]. Accordingly, the combination of RNAi against cellular and viral targets considerably enhances the antiviral effect [11].

Here, we have characterized particular responses to the inhibition of HIV-1 infection by shRNA-mediated CCR5 downregulation and the effect of a treatment combined with inhibition of viral replication by shRNA against the viral *rev* gene.

## Materials and methods

### Plasmid construction

shRNA coding sequences (CCR5, 5′-caggttggaccaagctatg-3′; Rev, 5′-acttactcttgattgtaac-3′; GFP, 5′-gaacggcatcaaggtgaac-3′) were cloned into the pRetroSuper (pRS), pSUPER (pSR) [12] (kindly provided by Dr R. Agami; The Netherlands Cancer Institute) or pCIneo vectors (Promega Corp., Madison WI). The plasmids pRS-shCCR5 (shCCR5), pSR-shRev and pRS-shGFP (shGFP) have been described previously [3]. The pCIneo-shRev (shRev) construct was generated by transferring the full shRNA transcription unit from the pSR-shRev vector into the XhoI and AccI sites of the pCIneo plasmid. The efficiency of this vector, evaluated by transient co-transfection with the Rev-GFP vector into HeLa T4 cells, was similar to the result previously observed for the pSR-shRev vector [3]. The Rev-GFP vector was constructed by removing the *rev* cDNA at the SnaBI and SspI sites from the pcRev plasmid (kindly provided by Prof. Bryan Cullen; Duke University Medical Centre, Durham NC) and cloning the segment into the Eco47III and SmaI sites of the pEGFP-N3 vector.

### Cell lines and transfections

MAGI-CCR5 cells [13] kindly provided by Prof. Jan Andersson (Karolinska University Hospital, Huddinge Sweden) were maintained in DMEM containing 300 µg G418 (geneticin, Invitrogen, Carlsbad CA), 1 µg puromycin (Sigma-Aldrich, Sweden) and 100 µg hygromycin B (Calbiochem, San Diego CA) per ml. The human U937 cells (DSMZ ACC 5, Braunschweig Germany) were cultured in RPMI medium. To enhance CCR5 expression, these cells were maintained with 1 µM retinoic acid (RA; Sigma, Sweden) for one week prior to the experiments [14]. All media were supplemented with 10% FBS and 50-µg gentamicin per ml. All cells were cultured at 37^o^C and 5% CO_2_. Culture media, FBS and gentamicin were purchased from Gibco (Invitrogen). For transient transfection, MAGI-CCR5 cells were transfected with 2 µg of shRev, shCCR5 or shGFP DNA using the FuGENE 6 reagent, following the manufacturer’s recommendations (Roche Applied Science, Sweden). U937 cell lines stably expressing shCCR5 (U937-shCCR5), shRev (U937-shRev) or shGFP (U937-shGFP) plasmids, were established by transfection by electroporation (20 µg DNA, 0.5 ml FBS-free medium, 1 × 10^7^ cells, 0.4 cm gap cuvette, exponential decay protocol of 320 V and 950 µF pulse) in the Gene Pulser XCell Electroporation System (Bio-Rad, Hercules CA). Transfectants were selected with 2 µg puromycin or 800 µg geneticin per ml. U937 cells stably expressing both, shCCR5 and shRev (U937-shCCR5-Rev) were established by transfecting U937-shCCR5 cells with shRev DNA, using the above-mentioned protocol; transfectants were selected with both puromycin and geneticin. Human PBMC from three donors were separated from whole blood by centrifugation on Ficoll-Paque (GE Healthcare, Uppsala Sweden). Cells were cultured in RPMI GlutaMax (Gibco Life Sciences, Paisley Scotland) containing 200 IU/ml rIL-2, 5 µg/ml PHA (GE Health Sciences), 1% gentamicine, 1% non-essential amino acids, 20 mM HEPES (Gibco Life Sciences) and 10% inactivated human AB + serum. Cell cultures were maintained at 37^o^C and 5% CO_2_ for 72 h, prior to infection.

### Western blotting

For CCR5 immunoblot, MAGI-CCR5 cells were transfected with shCCR5, harvested after 48 h and treated with lysis buffer. Proteins were denatured by heating to 65^o^C, resolved by 7% SDS-PAGE and electro-transferred onto nitrocellulose membranes. The CCR5 protein was detected with the goat polyclonal antibody CKR-5 (1:200; C-20; Sta Cruz Biotechnology Inc., Sta Cruz CA) and a secondary HRP-conjugated polyclonal rabbit anti-goat IgG antibody (1:2000; DAKO, Denmark). Quantification of the bands intensity was performed by the Image Studio Lite software (Licor Inc. US). As background, a manually selected region on the protein track of HeLa cells was used.

### HIV-1 infection

MAGI-CCR5 cells were transfected with shCCR5 or shGFP plasmids and infected after 48 h with the primary isolate HIV-1 6920 clade B (HIV-1 6920B) [15]. Cells were propagated for 9 days and supernatants (approximately 50%) were collected and replaced with fresh medium every third day and HIV-1 p24 antigen concentration was measured by capture ELISA assay [16].

RA-stimulated U937 cells stably expressing the different shRNAs and/or PBMC were seeded in 24-well plates (5 × 10^5^ cells/well). After 12 h incubation, the medium was removed and cells were infected with supernatants containing different TCID_50_ of HIV-1 6920B. The cells were incubated for 24 h and shaken every 30 min for the first 4 h. Then, they were washed to remove excess virus and fresh medium was added. The medium was collected and replaced every 72 h and the concentration of HIV-1 p24 antigen was measured in the supernatants.

The kinetics of the inhibitory effect of viral replication on titration experiments of U937-shCCR5 or U937-shRev cells mixed at various proportions with U937-shGFP cells was evaluated on 5 × 10^5^ cells of each mixture (400/0, 300/100, 200/200, 100/300 and 0/400 ul from a suspension of 1.25 × 10^6^ cells/ml of shRNA-protected/-unprotected cells), seeded in a 24-well plate. Cells were challenged with the HIV-1 6920B as described above and cultured for 13–14 days. The p24 production was measured in supernatants on days 3, 7, 10 and 13/14 days.

### Statistical analysis

Comparison of the different shRNA treatments efficiency on the inhibition of viral replication was performed by two-way ANOVA and Tukey or Bonferroni post hoc (95% CI); data are presented as mean values (± SD) of two replicates from one of two similar experiments. The kinetics of HIV-1 p24 antigen production by U937-shCCR5 and U937-shRev cell cultures were evaluated by comparative nonlinear regression analysis (exponential growth model). The inhibitory effect on viral p24 production in titration experiments of HIV-1 infection of shRNA-protected (U937-shCCR5 or U937-shRev) cells mixed with different proportions of unprotected (U937-shGFP) cells, harvested at different timepoints, was initially evaluated by single curve regression analysis for each timepoint dataset. Then, the fit pattern trend of each curve, was visually inspected. Since individual regression analysis of each timepoint dataset, represents assays under different conditions, which affect the shape of the kinetics curves trend, a global fitting model was chosen as the most suitable approach for simultaneous analysis of the multiple datasets acquired under different experimental conditions [17]. A global nonlinear regression analysis (simultaneous multicurve nonlinear least-squares) with an asymmetrical profile likelihood confidence interval was used to find a global fitting curve that better defines each family of datasets, with 95% CI, between the range of growing timepoint conditions. The kinetics of the inhibition experiments were fit both to an exponential plateau and to a line global model for the dataset family of U937-shCCR5 or U937-shRev cell cultures. The two models were compared by the Akaike’s Information Criterion (AICc) method to determine the relative likelihood of each being correct.

Statistical analyses and charts were processed with GraphPad Software (San Diego, CA).

## Results and discussion

**Fig. 1:**
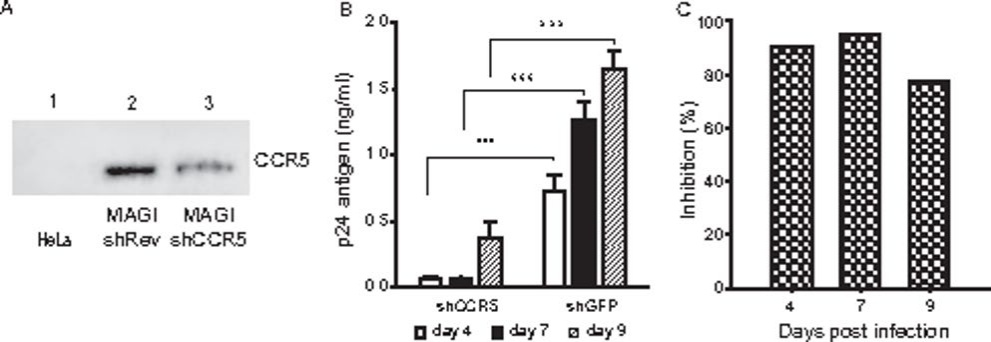
*shRNA-mediated downregulation of CCR5 expression* (A) CCR5 immunoblot of protein extracts from HeLa cells (line 1; I: 0.00 background) and from MAGI-CCR5 cells transfected with shRev (line 2; control plasmid; I: 6.86 × 10^6^) or with shCCR5 (line 3; I: 3.66 × 10^6^). (B) HIV-1 replication (mean ± SD, 95% CI; *, *p* < 0.05; **, *p* < 0.01; ***, *p* < 0.005; two-way ANOVA and Tukey multiple comparison test) and (C) viral infection inhibition percent, on MAGI-CCR5 cells transiently expressing shCCR5 or shGFP challenged with HIV-1 6920B (20 TCID_50_) and cultured for 9 days. I: signal intensity corrected for background

The efficiency of shCCR5 was initially evaluated in MAGI-CCR5 cells. Transient expression of shCCR5 in MAGI-CCR5 cells induced downregulation of the CCR5 coreceptor to 47% (Fig. 1 A), similar to the results of our previous report using U937 cells [3]. MAGI-CCR5 cells transiently expressing shCCR5 or shGFP were then challenged with HIV-1 6920B and viral p24 antigen was analysed in the supernatants by the ELISA capture assay. Assay of HIV-1 p24 concentration was selected as an estimate of viral replication by practicality since new capsid antigens are formed during viral replication. In the shCCR5-protected culture, a significantly lower production of the p24 antigen was observed on days 4, 7 and 9 after infection (*p* = 0.0043, *p* < 0.0002 and *p* < 0.0001, respectively) compared with that in the unprotected culture (Fig. 1B) and the inhibition of viral replication was 91, 95 and 77% on the corresponding days (Fig. 1 C); these results suggest that HIV-1 infection requires a minimal threshold concentration of CCR5 on the cell surface and that even a partial reduction in CCR5 expression substantially decreases HIV-1 infection. The threshold of surface CCR5 density required for HIV-1 infection has also been shown in previous in vitro studies and in vivo in individuals heterozygous for CCR5Δ32 [15, 18].

**Fig. 2:**
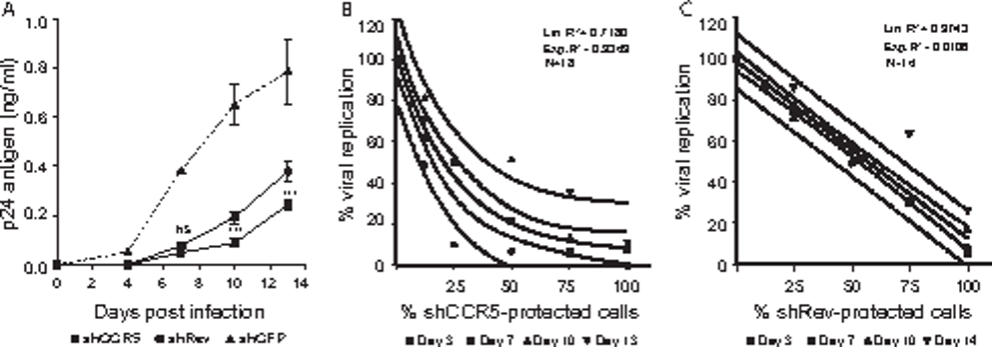
*Inhibitory effect of shCCR5 and shRev on HIV-1 infection and replication over time, and on a mixture of shRNA- protected and unprotected cell cultures* (A) p24 antigen production on U937-shCCR5 (shCCR5) and U937-shRev (shRev) cells challenged with 100 TCID_50_ of HIV-1 6920B and cultured for 13 days (mean ± SD, 95% CI, on days 4, 7, 10 and 13 after infection; NS, not statistically significant; *, *p* < 0.05; **, *p* < 0.01; ***, *p* < 0.005; two-way ANOVA and the Bonferroni test) and kinetics of viral replication (comparative nonlinear regression analysis, exponential growth model: p = 0.0089; k = 0.2246 and 0.2612 and doubling time = 3.086 and 2.654 for shCCR5 and shRev cultures, respectively); U937-shGFP (shGFP) cells used as control. (B and C) Global fitting curves from a nonlinear regression analysis for a family of datasets of viral replication of cultures grown for 3, 7, 10 and 13/14 days of (B) U937-shCCR5 or (C) U937-shRev cells mixed at different proportions with U937-shGFP cells and challenged with HIV-1 6920B (100–150 TCID_50_). Data represent % viral replication with respect to starting value with titration type. Solid line: best fitting curve models (line or exponential plateau) based on the Akaike´s Information Criterion; inner dashed lines: 95% CI (asymmetrical) between the range of growing timepoint conditions (U937-shCCR5 intercepts at X = 0: 114.8 and 92.58, at X = 100: 16.73 and − 4.081; U937-shRev intercepts at X = 0: 103.2 and 94.06); outer dashed lines: 95% CI prediction bands

Then, the efficiency of shCCR5 and shRev was evaluated in the U937 cells stably expressing one of these vectors (Fig. 2 A). When U937-shCCR5 or U937-shRev cells were infected with HIV-1 6920B (100 TCID_50_) for two weeks, the comparative nonlinear regression analysis (exponential growth) of the viral replication kinetics showed an overall lower replication in the shCCR5-modified cells than that in the shRev-modified cells (95% CI; *p* = 0.0089; k = 0.2246 and 0.2612 and doubling time = 3.086 and 2.654 for shCCR5 and shRev cultures, respectively). The p24 concentration in the supernatants of the two cell cultures at various timepoints showed significantly lower production by the U937-shCCR5 culture on days 10 and 14 (*p* = 0.0042 and *p* = 0.0007, respectively) indicating a more sustained protective effect of the shCCR5 treatment (Fig. 2 A).

Prevention of viral entry in the cells or blocking viral replication in infected cells may have a distinct protective effect in vivo because shRNA delivery can protect only a certain fraction of HIV-1 target cells. In this scenario, blocking viral infection by downregulating the CCR5 coreceptor rather than by inhibiting replication by targeting viral genes may provide a better selective survival and proliferative advantage to the modified cells by reducing the HIV-1 cytopathogenic effect mediated by the coreceptor engagement [19]. Targeting only viral genes may block replication but does not necessarily block infection; thus, the proportion of unprotected-infected cells and the production of viral particles by these cells may substantially influence the infection, proliferation and survival rate of the shRev-protected cells. Since U937-shCCR5 and U937-shRev cells are models of viral entry and inhibition of early viral replication, respectively, the kinetics of the inhibitory effect on viral p24 production in these cells were evaluated in titration experiments of HIV-1 infection of shRNA-protected (U937-shCCR5 or U937-shRev) cells mixed with various proportions of unprotected (U937-shGFP) cells (Fig. 2B C).

Interestingly, a different dynamic of viral replication inhibition was observed in the two cultures. The global curve fitting by nonlinear regression analysis that defines the family of the datasets of viral replication in the U937-shCCR5/U937-shGFP cell cultures grown for 3, 7, 10 and 13 days, showed a better fit to a nonlinear exponential plateau than to a line model (AICc = 93.90 and 130.2 for exponential plateau and line fit models, respectively; evidence ratio = 7.6 × 10^7^). As shown in Fig. 2B C, the curve of the U937-shCCR5 cell cultures displayed saturation at approximately 75–80% of protected cells, whereas the curve of the U937-shRev cells showed an almost linear decrease of viral replication with respect to the percentage of shRev-transfected cells (AICc = 62.18 for line model; results of calculations for the exponential plateau model return “ambiguous”). In the U937-shCCR5 cell experiment, a lower number of shCCR5-modified cells conferred a higher inhibition of viral replication than that in the cultures of the U937-shRev cells. Cultures containing as low as 25% of U937-shCCR5 cells were characterized by viral replication inhibition of approximately 50%, while the cultures with 50 and 75% of shCCR5-modified cells were characterized by inhibition by approximately 75% and 90%, respectively. This observation is very relevant for the outcome of RNAi treatment of HIV-1 infection expected in a clinical setting, since it shows that in a model of cell populations composed of shRNA-protected and unprotected cells, which may happen in vivo, shCCR5 has a better protective potential than shRev against CCR5-utilizing HIV-1. In this situation, the extent of HIV-1 infection and cytopathogenic effect will depend on the number of unprotected and infected cells. Theoretically, CCR5-negative cells will be less susceptible to the free virus, viral cell-to-cell infection [20] and coreceptor-induced alterations in signal transduction and cytopathogenic effects [21]. Thus, apoptosis of infected cells will decrease the viral production and therefore, the number of shCCR5-expressing cells may become higher than the number of shRev-expressing cells in the corresponding cultures because of a higher survival and proliferative advantage. These distinctive responses to the inhibition of HIV-1 infection by shRNA CCR5 downregulation reinforce that the CCR5 coreceptor can be considered a strategic target for HIV-1 RNAi therapy, which is in agreement with the previous results of in vitro and in vivo studies [22, 23]. However, due to the virus´s ability to switch its coreceptor affinity (e.g., CXCR4 or CXCR6) [9, 24], a strong inhibition of viral replication using RNAi against other suitable viral targets in combination with inhibition of *CCR5* may enhance the protective effect against highly infectious doses and may reduce the rate of escape mutant generation.

**Fig. 3:**
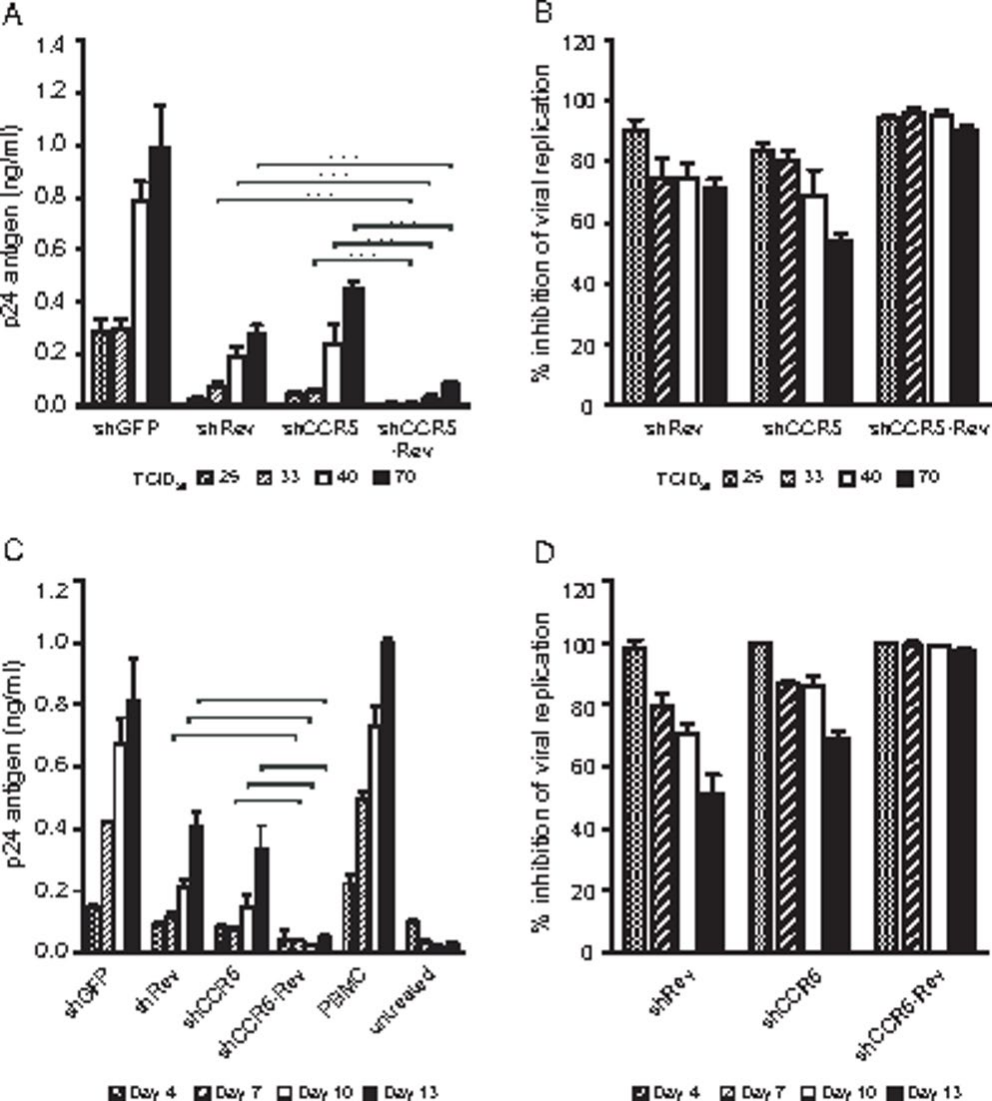
*Enhanced and sustained protection against HIV-1 infection by simultaneously targeting CCR5 and rev transcripts* U937-shCCR5 (shCCR5), U937-shRev (shRev), U937-shCCR5Rev (shCCR5-Rev) and U937-shGFP (shGFP) cells were infected with HIV-1 6920B at 25–70 TCID_50_ (**A** and **B**) or 100 TCID_50_ (**C** and **D**); p24 concentration was measured in supernatants on days 4 (**A** and **B**) and 4, 7, 10 and 14 (**C** and **D**). Data are (**A** and **C**) HIV-1 p24 concentration and (**B** and **D)** % inhibition of viral replication with respect to that of the U937-shGFP cells (mean ± SD, 95% CI, two-way ANOVA and the Bonferroni test). PBMC, infection control; untreated, uninfected shGFP cells; ***, *p* < 0.005

Accordingly, we demonstrate that when cellular and viral genes are simultaneously targeted by the two shRNAs, the protection against HIV-1 infection is bolstered and the combined protective properties of these shRNAs are enhanced (Fig. 3). In cultures of U937-shCCR5, U937-shRev and U937-shCCR5-Rev cells infected with increasing doses of HIV-1 6920B for four days, double-transfected cells have significantly lower viral replication than the cells transfected with shCCR5 or shRev (*p* < 0.0001 and *p* < 0.0001, respectively) according to comparison at increasing infective doses of 40 and 70 TCID_50_ (Fig. 3 A). The combined protective effect of the shCCR5-Rev treatment was higher than any single treatment and ranged from 95 to 98% of viral replication inhibition at increasing infective doses within the limits of 25–70 TCID_50_ (*p* = 0.0040 and 0.0003, < 0.0001 and 0.0006, < 0.0001 and < 0.0013 for shCCR5 and shRev comparisons with shCCR5-Rev at 33, 40 and 70 TCID_50,_ respectively). In contrast, the protection by cells transfected with a single construct was markedly exceeded by increased viral infectious doses (Fig. 3B).

Furthermore, when different cell lines were challenged with a high viral dose (100 TCID_50_) and cultured for 14 days, the double-transfected cells showed a significantly lower viral replication over the whole experimental period compared with that in the shCCR5- or shRev-modified cells (p = 0.0037 and < 0.0001, < 0.0001 and < 0.0001 for shCCR5 and shRev comparison with shCCR5-Rev at 10 and 13 days, respectively. The levels of viral replication over time in the U937-shGFP-infected cultures were similar to those in infected human PBMC (Fig. 3 C). The cell lines expressing both shRNAs showed a higher and more sustained inhibition of p24 production (100%-98%) than the cells modified with a single vector over the whole culture period (*p* = 0.0065 and < 0.0001, 0.0044 and < 0.0001, < 0.0001 and < 0.0001, for shCCR5 and shRev comparison with shCCR5-Rev at 7, 10 and 14 days, respectively. The inhibitory effect of the cells modified by a single vector declined after day 7 (Fig. 3D). These results are in agreement with previous in vitro and in vivo studies on simultaneous targeting of *CCR5* and various viral genes by RNAi [22, 23].

In summary, the present study demonstrates that shRNAs targeting the *CCR5* or *Rev* transcripts have a distinct impact on HIV-1 infection and that shCCR5 has additional advantages. High protective effect of partial downregulation of CCR5 expression, better performance of shCCR5 in a model of cell population composed of shRNA-protected and unprotected cells, and better sustained functionality account for efficient inhibition of HIV-1 infection by shCCR5. On the other hand, inhibition of viral replication by shRev was close to 100% although the effect was less sustained. Thus, simultaneous treatment with both shRNAs resulted in a clearly enhanced and more stable viral replication inhibition. Both properties are essential for delay or prevention of the appearance of RNAi virus escape mutants or switch of the coreceptor affinity.

Congenital absence of CCR5 appears to be well tolerated [25]; thus, considering all previous studies [26], the results of the present study reinforce the convenience of inclusion of CCR5 inhibitors in HIV-1 gene silencing treatment schedules to further reduce endogenous viral infection and replication in a clinical setting in a properly ART-treated HIV-1 infected individual.
